# Trends in the Use of Buprenorphine in US Emergency Departments, 2002-2017

**DOI:** 10.1001/jamanetworkopen.2020.21209

**Published:** 2020-10-20

**Authors:** Taeho Greg Rhee, Gail D’Onofrio, David A. Fiellin

**Affiliations:** 1 Department of Public Health Sciences, School of Medicine, University of Connecticut, Farmington; 2 Department of Psychiatry, Yale School of Medicine, New Haven, Connecticut; 3 Department of Emergency Medicine, Yale School of Medicine, New Haven, Connecticut; 4 Program in Addiction Medicine, Yale School of Medicine, New Haven, Connecticut; 5 Department of Health Policy and Management, Yale School of Public Health, New Haven, Connecticut; 6 Department of Internal Medicine, Yale School of Medicine, New Haven, Connecticut

## Abstract

This cross-sectional study examines the trends in initiation of buprenorphine treatment for opioid use disorder during emergency department visits in the United States.

## Introduction

Buprenorphine is an effective treatment for opioid use disorder, and its use has increased over time in outpatient settings^1^ and among commercially insured adults in the United States.^2^ Although the American College of Emergency Physicians and other professional organizations have advocated for buprenorphine treatment initiation in emergency department (ED) settings,^3^ little is known about how often this practice occurs in EDs nationwide. We examined the trends of buprenorphine use in EDs in the United States from 2002 (when it was approved) through 2017.

## Methods

This cross-sectional study used 2002-2017 data from the National Hospital Ambulatory Medical Care Survey, which provides nationally representative samples of ED visits.^4^ We examined ED visits by patients aged 18 years or older. From our examination of these visits, we estimated trends of visits during which buprenorphine was dispensed using generic names (ie, buprenorphine or buprenorphine-naloxone). We combined data into 2-year intervals to improve the stability of our estimates.^4^ We quantified the number of visits during which these medications were used per 100 000 ED visits. This study was deemed to be exempt from human subjects review by the Yale School of Medicine’s Institutional Review Board because we used publicly available, deidentified data. Study procedures followed the Strengthening the Reporting of Observational Studies in Epidemiology (STROBE) reporting guideline for cross-sectional studies.

We compared the proportions of ED visits during which buprenorphine was dispensed between 2002-2003 and 2016-2017 using an adjusted Wald χ^2^ test. To test linear trends over time, we transformed the survey year range from 0 (for 2002-2003) to 1 (for 2016-2017). Odds ratios associated with this transformed variable represent change in the odds of proportion of medication use during ED visits across the entire study period. We further conducted subgroup analyses by age, sex, race/ethnicity, and region. Stata, version 15.1 MP/6-Core (Stata Corp) was used for all analyses, and we used the *svy* commands in that statistical software to account for multiple years and the complex survey sampling design of the National Hospital Ambulatory Medical Care Survey, including unequal probability of selection, clustering, and stratification.^4^ Two-sided *P* < .05 was used to test statistical significance.

## Results

Between 2002 and 2017, there were 441 475 ED visits during which buprenorphine was dispensed. Among the patients who received buprenorphine, the mean (SD) age was 39.1 (14.8) years, 49.1% were male, 66.3% were non-Hispanic White, 31.0% were non-Hispanic Black, and 92.7% were from urban areas. The use of buprenorphine increased from 12.3 per 100 000 ED visits in 2002-2003 to 42.8 per 100 000 ED visits in 2016-2017 (odds ratio for linear trend, 3.31; 95% CI, 1.04-10.50) (Figure). From 2002-2003 to 2016-2017, the use of buprenorphine increased linearly among individuals aged 19 to 44 years (from 10.4 to 38.4 per 100 000 ED visits; *P* = .02). The use of buprenorphine also increased over time in the Northeast (from 0.0 to 8.2 per 100 000 ED visits; *P* = .01) and metropolitan areas (from 12.2 to 42.8 per 100 000 ED visits; *P* = .03) (Table).

**Figure.**
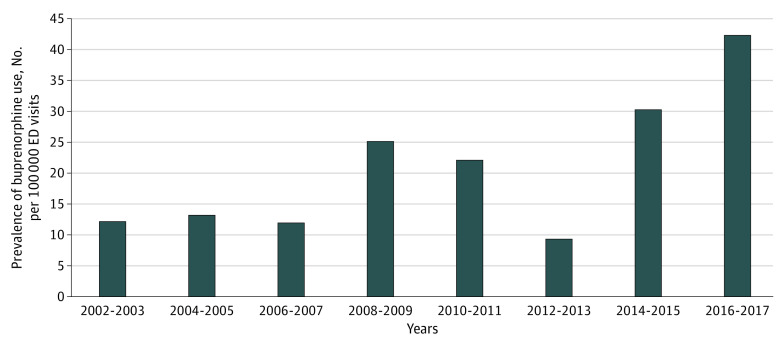
Trends in the Use of Buprenorphine in Emergency Departments (EDs) per 100 000 Visits in the United States, 2002-2017 Data are from National Hospital Ambulatory Medical Care Survey. Estimates are adjusted to annual ED visits. Buprenorphine use increased significantly from 2002-2003 to 2016-2017 (odds ratio for linear trend, 3.31; 95% CI, 1.04-10.50; *P* = .04).

**Table.** Trends in the Use of Buprenorphine in EDs by Demographic Factor in the United States, 2002-2017^a^

Demographic factor	Prevalence of buprenorphine use, No. per 100 000 ED visits								2002-2003 vs 2016-2017		*P* value for linear trend
	2002-2003	2004-2005	2006-2007	2008-2009	2010-2011	2012-2013	2014-2015	2016-2017	*F* statistic	*P* value	
Total ED visits	224 057 757	225 539 223	235 993 594	259 833 549	266 139 777	261 222 653	278 363 641	284 568 568			
Buprenorphine use											
Overall visits	12.3	13.3	12.1	25.2	22.3	9.4	30.2	42.8	5.37	.02	.04
Opioid-related visits	1.9	0.0	0.0	4.0	0.0	0.8	2.2	1.5	1.65	.20	.32
Age, y											
≤18	0.0	1.9	0.0	0.0	1.0	0.0	0.0	0.0	NC^b^	NC^b^	.21
19-44	10.4	6.0	7.5	7.5	16.7	9.4	20.4	38.4	5.31	.02	.02
45-64	1.3	2.4	4.5	11.5	3.6	0.0	9.8	4.4	1.59	.21	.55
≥65	0.6	3.1	0.0	6.2	1.0	0.0	0.0	0.0	0.11	.74	.03
Sex											
Male	6.4	4.5	5.8	13.6	11.5	5.9	16.1	18.5	1.80	.18	.12
Female	5.9	8.8	6.3	11.7	10.8	3.6	14.1	24.3	3.43	.06	.17
Race/ethnicity											
Non-Hispanic White	9.0	9.7	4.9	14.6	19.2	9.4	24.5	20.1	1.46	.23	.06
Other	3.3	3.6	7.2	10.6	3.1	0.0	5.8	22.7	3.98	.046	.24
Region											
Northeast	0.0	0.0	0.9	5.8	8.0	5.3	3.8	8.2	NC^b^	NC^b^	.01
Midwest	0.1	1.9	6.2	6.0	4.4	2.5	3.0	1.9	4.09	.04	.94
South	12.2	11.4	4.9	13.5	7.7	0.0	21.6	20.4	0.75	.39	.31
West	0.0	0.0	0.0	0.0	2.1	1.6	1.8	12.3	NC^b^	NC^b^	.08
Metropolitan statistical area											
Yes	12.2	10.3	12.1	18.2	21.4	2.5	29.1	42.8	5.39	.02	.03
No	0.1	3.1	0.0	7.1	0.9	0.0	1.1	0.0	2.46	.12	.22

Abbreviations: EDs, emergency departments; NC, not calculable.

^a^ Data are from National Hospital Ambulatory Medical Care Survey. Estimates are adjusted to annual ED visits.

^b^ Insufficient sample sizes to perform a Wald χ^2^ test for comparison.

## Discussion

Buprenorphine use increased in EDs between 2002 and 2017, the years for which the most recent data are currently available. The increase in overall buprenorphine use could be attributed to an increase in opioid-related ED visits.^5^ Limitations include the assumption that all buprenorphine was provided for opioid use disorder and the lack of information on dosing, route of administration, formulation, or prescriptions written for ongoing treatment after ED discharge. It appears that the sampling strategy or data collection method in 2012-2013 was different from that used in other years because less buprenorphine use was captured.

Despite the limitations, the present study found increased use of buprenorphine in ED settings, a promising strategy for narrowing the treatment gap. Potential barriers to ED adoption of buprenorphine treatment initiation for untreated opioid use disorder have been identified,^6^ and research on strategies to address these should be prioritized.
